# GABA-Glycine Cotransmitting Neurons in the Ventrolateral Medulla: Development and Functional Relevance for Breathing

**DOI:** 10.3389/fncel.2019.00517

**Published:** 2019-11-19

**Authors:** Johannes Hirrlinger, Grit Marx, Stefanie Besser, Marit Sicker, Susanne Köhler, Petra G. Hirrlinger, Sonja M. Wojcik, Volker Eulenburg, Ulrike Winkler, Swen Hülsmann

**Affiliations:** ^1^Carl-Ludwig-Institute for Physiology, Faculty of Medicine, University of Leipzig, Leipzig, Germany; ^2^Department of Neurogenetics, Max-Planck-Institute for Experimental Medicine, Göttingen, Germany; ^3^Medizinisch-Experimentelles Zentrum, Faculty of Medicine, University of Leipzig, Leipzig, Germany; ^4^Department of Molecular Neurobiology, Max-Planck-Institute for Experimental Medicine, Göttingen, Germany; ^5^Department for Anesthesiology and Intensive Care Therapy, Faculty of Medicine, University of Leipzig, Leipzig, Germany; ^6^Department of Anaesthesiology, University Medical Center, Georg-August University, Göttingen, Germany; ^7^Center for Nanoscale Microscopy and Molecular Physiology of the Brain, Göttingen, Germany

**Keywords:** GABA, glycine, cotransmission, ventrolateral medulla, preBötzinger complex, neuronal control of breathing, split-Cre

## Abstract

Inhibitory neurons crucially contribute to shaping the breathing rhythm in the brain stem. These neurons use GABA or glycine as neurotransmitter; or co-release GABA and glycine. However, the developmental relationship between GABAergic, glycinergic and cotransmitting neurons, and the functional relevance of cotransmitting neurons has remained enigmatic. Transgenic mice expressing fluorescent markers or the split-Cre system in inhibitory neurons were developed to track the three different interneuron phenotypes. During late embryonic development, the majority of inhibitory neurons in the ventrolateral medulla are cotransmitting cells, most of which differentiate into GABAergic and glycinergic neurons around birth and around postnatal day 4, respectively. Functional inactivation of cotransmitting neurons revealed an increase of the number of respiratory pauses, the cycle-by-cycle variability, and the overall variability of breathing. In summary, the majority of cotransmitting neurons differentiate into GABAergic or glycinergic neurons within the first 2 weeks after birth and these neurons contribute to fine-tuning of the breathing pattern.

## Introduction

Inhibitory neurons represent a large proportion of neurons in the respiratory network in the ventrolateral medulla. These neurons are distributed in the ventral respiratory column including the area of Bötzinger complex and the preBötzinger complex (preBötC). However, the function of inhibitory neurons in general and specifically to which aspects of breathing regulation these cells contribute to remains controversial. Certainly, inhibitory neurons are required for adaptation of the respiratory network to internal and external environmental changes. Here, these neurons mediate the negative feedback from central and peripheral sensors, e.g., in the Breuer-Hering reflex (Ezure and Tanaka, 2004). Moreover, these neurons are important for phase transition from inspiration to expiration similar to phase transitions in other motor systems (Busselberg et al., 2001; Richter and Smith, 2014; Hülsmann et al., 2019). In contrast, it has remained a matter of intense debate whether inhibitory neurons serve a vital function in the generation of the respiratory rhythm (Janczewski et al., 2013; Sherman et al., 2015; Marchenko et al., 2016; Cregg et al., 2017; Ausborn et al., 2018; Baertsch et al., 2018).

Inhibitory neurons come in different flavors with respect to the neurotransmitter they use. The two major inhibitory neurotransmitters are GABA and glycine. However, in addition to GABAergic and glycinergic neurons, also GABA-glycine cotransmitting neurons (GGCN) were identified in the ventral respiratory column (Koizumi et al., 2013; Rahman et al., 2013, 2015), as well as in other brain areas (Triller et al., 1987; Spike et al., 1993; Jonas et al., 1998; Wu et al., 2002; Nerlich et al., 2014, 2017). The functional relevance of this cotransmission is still unclear (Burnstock, 2004). It has been suggested that (1) simultaneously released transmitters might act on different post-synaptic terminals; (2) one transmitter might feedback on the presynaptic terminal to modulate transmitter release; (3) both transmitters might act synergistically on the same post-synaptic terminal or (4) they provide a negative cross talk on post-synaptic receptors (Burnstock, 2004). In the case of GABA and glycine, the different kinetics of transmitter evoked currents of GABA_A_-receptors (slower) and glycine receptors (faster) might shape the synaptic response of the post synaptic neuron (Jonas et al., 1998; O’Brien and Berger, 1999; Keller et al., 2001; Russier et al., 2002; Rahman et al., 2013; Nerlich et al., 2014). GABA and glycine are filled into synaptic vesicles by the same transporter, i.e., vesicular inhibitory amino acid transporter (VIAAT, also called VGAT, official gene symbol Slc32a1) and the content of GABA and glycine of an individual synaptic vesicle can be modified by the presynaptic neuron (Wojcik et al., 2006; Aubrey, 2016). Therefore, the presynaptic neuron might fine-tune the kinetics of the synaptic response of the post synaptic neuron by varying the ratio of GABA and glycine within the synaptic vesicles (Jonas et al., 1998). However, the precise physiological function and relevance of GABA and glycine cotransmission has remained unclear.

The occurrence of GGCN has been associated with immature neuronal networks, since in many instances the number of GGCN is reduced during maturation of synapses, neurons and neuronal networks (Aubrey and Supplisson, 2018). Consistently, GGCN were often detected in young animals (Jonas et al., 1998; O’Brien and Berger, 1999; Russier et al., 2002; Rahman et al., 2013) and developmental shifts from GGCN to an either GABAergic or glycinergic phenotype have been described in a number of neuronal networks (Gao et al., 2001; Keller et al., 2001; Nerlich et al., 2017). Similarly, in the preBötC almost 70% of inhibitory neurons show a GGCN phenotype during late embryonic development, and their number appears to decrease just around birth (Rahman et al., 2015). However, the detailed time course of this transition during postnatal development as well as the cellular relationship of early GGCN and later GABAergic or glycinergic neurons has not been elucidated so far.

Mice with impairment of glycinergic neurotransmission compensate this deficit rather well at birth (Büsselberg et al., 2001; Zhao et al., 2006; Hülsmann et al., 2019), while mice with loss of synaptic inhibition involving both inhibitory neurotransmitters due to the lack of VIAAT are not viable (Wojcik et al., 2006; Fujii et al., 2007). Double knock out of both glutamate decarboxylase isoforms (Gad1, Gad2; also known as GAD67 and GAD65, respectively) also leads to lethality at birth (Fujii et al., 2007). It is important to note that until today no mouse model with a complete loss of only glycinergic transmission and unaltered GABAergic transmission is available. Interestingly, conditional knockout of the Viaat gene using a glycine transporter 2 (Glyt2; official gene symbol Slc6a5)-Cre driver line (thereby targeting neurons with a glycinergic, but not necessarily a pure glycinergic phenotype; Ishihara et al., 2010) leads to a complete loss of not only glycinergic inhibitory post-synaptic currents (IPSC) but also eliminates GABAergic IPSC in the brain stem slice preparation (Rahman et al., 2015). This finding suggests that many GABAergic neurons also activate the transgene specific for glycinergic neurons, Glyt2-Cre, at some point during development, most likely during a period when a cotransmitting phenotype was expressed (Rahman et al., 2015).

In this study we present the developmental phenotypic profile of inhibitory neurons in the preBötC as well as the cellular relationship of early GGCN and later GABAergic or glycinergic cells. To label GGCN neurons irreversibly and to track their fate we developed novel transgenic mouse lines based on the split-Cre and split-CreERT2 system (Hirrlinger et al., 2009a, b), allowing us to specifically target GGCN genetically. We find that most inhibitory neurons in the ventrolateral medulla are GGCN first and later develop into either a GABAergic or a glycinergic phenotype. Furthermore, we functionally inactivated GGCN neurons by selective deletion of the Viaat gene in this cell population, which resulted in changes of the precision of the breathing pattern.

## Materials and Methods

### Ethics Statement

In accordance with the guidelines for the welfare of experimental animals issued by the European Communities Council Directive (2010/63/EU) and with the German Protection of Animals Act (Tierschutzgesetz), mice were bred in the animal facilities of the Medical Faculty of the University of Leipzig and of the University Medical Center Göttingen. Mice were housed in individually ventilated cages in a specific pathogen free environment in a 12 h/12 h light dark cycle with access to food and water *ad libitum*. Experiments were approved by the animal welfare office of the Faculty of Medicine, University of Leipzig and the governmental authorities of Saxony (Landesdirektion Sachsen, registration number TVV66/12) or by the animal welfare office and commission of the University Medical Center Göttingen and the respective governmental authorities of Lower Saxony (LAVES, registration number T12/11). This article does not contain any studies with human participants performed by any of the authors. Therefore, no informed consent was needed.

### Transgenic Mice

The COFLUOR mouse line harboring the Glyt2-EGFP- (Zeilhofer et al., 2005) and Gad2-tdTomato alleles (Table 1) were described previously [C57BL/6J-Tg(Gad2-tdTomato)DJhi; RRID:IMSR_EM:10422; Besser et al., 2015]. Mouse lines expressing the split-Cre- or split-CreERT2-system were generated based on bacterial artificial chromosomes (BACs), clone RPCI23-407K8 containing the entire mouse Gad2 gene (Source Bioscience imaGenes, Berlin, Germany) or RP23-365E4 containing the whole mouse Glyt2 gene (official gene symbol Slc6a5) for targeting of GABAergic and glycinergic neurons, respectively.

**TABLE 1:** Mouse lines used in this study.

**Strain name**	**Transgenes**	**Gene symbol**	**Repository**	**Reference**
COFLUOR	Tg(Glyt2-EGFP)	Slc6a5		Zeilhofer et al., 2005
	Tg(Gad2-tdTomato)DJhi	Gad2	EM:10422	Besser et al., 2015
COTRANS	Tg(Glyt2-CCre)	Slc6a5	EM:12607	This work
	Tg(Gad2-NCre)	Gad2	EM:12607	This work
	Ai14 [Gt(ROSA)26Sor^tm 14(CAG–tdTomato)Hze^/J]	Rosa26	JAX 007914	Madisen et al., 2010
COTRIND	Tg(Glyt2-ERT2CCre)	Slc6a5	EM:12608	This work
	Tg(Gad2-NCreERT2)	Gad2	EM:12608	This work
	Ai14 [Gt(ROSA)26Sor^tm 14(CAG–tdTomato)Hze^/J]	Rosa26	JAX 007914	Madisen et al., 2010
VIACO	All alleles from COTRANS			
	ViaatFlx	Slc32a1		Rahman et al., 2015
indVIACO	All alleles from COTRIND			
	ViaatFlx	Slc32a1		Rahman et al., 2015

*EM: Strain number at the European Mouse Mutant Archive; JAX: Strain number at The Jackson laboratory. Strains EM:12607 and EM:12608 are currently in the process of deposition at the European Mouse Mutant Archive.*

To target GABAergic neurons with the split-Cre-system, either NCre or NCreERT2 (Hirrlinger et al., 2009a, b) were fused to an internal ribosome entry site (IRES), β-Galactosidase, a SV40-pA sequence and a FRT-flanked neomycin cassette. This construct was inserted by homologous recombination into BAC clone 407K8 at the ATG initiation codon of exon 1 of the Gad2 gene (Supplementary Figure 1).

To target glycinergic neurons, either the CCre or ERT2CCre open reading frames were cloned to an IRES, alkaline phosphatase, followed by a SV40-pA sequence and a FRT-flanked neomycin cassette. The targeting constructs were inserted into exon 2 of the Glyt2 gene within BAC clone 365E4 using homologous recombination as described previously (Zeilhofer et al., 2005; Ishihara et al., 2010).

Prior to injection into mouse oocytes, the neomycin cassette was removed from all BACs by Flp-mediated recombination (Supplementary Figure 1). BAC DNA was prepared using the NucleoBond BAC 100 Kit (Macherey-Nagel, Düren, Germany). Modified BACs were verified using PCR and sequence analysis. The successfully modified BACs were linearized using PI-SceI, purified using gel filtration and checked by pulse field gel electrophoresis. Pronuclear injection of linearized BAC-DNA into fertilized C57BL/6J oocytes was performed by the transgene core facility of the Max-Planck-Institute for Experimental Medicine (Göttingen, Germany). Founder mice were transferred to the animal facility of the Medical Faculty of the University of Leipzig. For Gad2-NCre and Glyt2-CCre one transgenic founder each was obtained and the lines were crossbred with each other and the Ai14-Cre reporter line expressing tdTomato after DNA recombination (Gt(ROSA)26Sor^TM 14(CAG–tdTomato)Hze^/J; Madisen et al., 2010). This triple transgenic mouse line is referred to as COTRANS-line (Table 1). For Gad2-NCreERT2 and Glyt2-ERT2CCre two transgenic founders each were obtained. One of these lines each were crossbred with the Ai14-Cre reporter line and used for the experiments of this study. This triple transgenic mouse line is referred to as COTRIND-line (Table 1).

Gad2-tdTomato mice [C57BL/6J-Tg(Gad2-tdTomato)DJhi] are available from the European Mouse Mutant Archive (RRID:IMSR_EM:10422). Gad2-NCre, Gad2-NCreERT2, Glyt2-CCre and Glyt2-ERT2CCre are currently deposited at the European Mouse Mutant Archive (strains EM:12607, EM:12608, Table 1). Ai14 mice are available from The Jackson Laboratory (strain 007914; Table 1). For obtaining Glyt2-EGFP and ViaatFlx mice the laboratories having generated these lines originally should be contacted.

### Genotyping

Genomic DNA from tail biopsies was isolated by boiling the tissue for 20 min in 25 mM NaOH. After neutralization with 40 mM TRIS/HCl, PCRs were performed using GoTaq (Promega, Mannheim, Germany) and the following primers (250 nM final concentration each; 5′–3′ sequence for forward/reverse primer given):

Gad2-NCre, Gad2-NCreERT2: CTCCCGCCACACGTACTC/CAAGTGATAATTTTTCGAAAGCAA;
Glyt2-CCre: GTGCCTGGACCTGGAATGTGTGC/CTCCAT CAGGGATCTGACTTGGTCAA;
Glyt2-ERT2CCre: TTTTGCACGAACTTGACATTG/TGGTA GGATCATACTCGGAATAGAG;
Gad2-tdTomato: GTGCAGGGTCGAGGCAAAGGCA/GGAC AGGATGTCCCAGGCGAAG;
Glyt2-EGFP: GCCGCTACCCCGACCAC/AGCATACGTGCA CCCGCCAGG;
Ai14 wt: CCTCCTGGCTTCTGAGGAC/AGGACAACGCCC ACACAC;
Ai14 tg: TACGGCATGGACGAGCTGTACAAGTAA/CATAG TTAAGAATACCAGTCAATCTT;
ViaatFlx wt: CCTTCTGGGTCCACACTTCTTCTA/CTCCCC GTCTTCGTTCTCCTCGTA;
ViaatFlx tg: CCTTCTGGGTCCACACTTCTTCTA/CTCCCCG TCTTCGTTCTCCTCGTA.

### Application of Tamoxifen

The time-controlled activation of the Cre recombinase was induced by application of 4-hydroxy-tamoxifen (Sigma-Aldrich, Munich, Germany). For mice at the age of p1 a stock solution of 20 mg/ml 4-OH-tamoxifen in ethanol was prepared. 100 μl 4-OH-tamoxifen stock solution were mixed with 500 μl sunflower oil, and 10 μl (0.066 mg) per 1 g body weight were injected intraperitoneal once a day on two consecutive days.

### Immunohistochemistry

Adult mice were transcardially perfused with 4% paraformaldehyde in phosphate buffered saline (PBS: 137 mM NaCl, 2.7 mM KCl, 8 mM Na_2_HPO_4_, 0.15 mM KH_2_PO_4_, pH 7.4). Mice were decapitated, the brain removed from the scull and post-fixed for 24 h in the same fixative. Younger animals were sacrificed by decapitation, while preparation of embryos was done as described (Rahman et al., 2015). Brains were subsequently fixed in 4% PFA/PBS for 24 h to 48 h. 45 μm thick, free-floating coronal sections of the brainstem were incubated with the primary antibodies. To identify GABAergic neurons a mixture of mouse anti-GAD2 antibody and mouse anti-GAD1 antibody was used, while glycinergic neurons were stained using rat anti-glycine antibody. Expression of split-Cre transgenes was verified using rabbit anti-ERα-, rabbit anti-β- Gal-, and mouse anti-Cre Recombinase antibodies (see Supplementary Table 1 for details on the antibodies). Sections were washed three times for 5 min in PBS and incubated with the secondary antibodies (Supplementary Table 1). After washing with PBS, sections were incubated in PBS containing 1 μg/ml DAPI (Roth, Karlsruhe, Germany) for 5 min to stain cell nuclei. Finally, sections were mounted in Immu-Mount (Thermo Scientific, Karlsruhe, Germany).

For quantification, all immunhistochemical experiments were performed on slices of at least three animals; for each animal, cells within three sections were counted. Confocal Images were acquired using a Zeiss LSM700 Axio Observer laser scanning microscope. In the ventrolateral medulla, slices were analyzed which contain the preBötzinger complex (Bregma −6.64 mm to Bregma −7.08 in adult mice; Paxinos and Franklin, 2001) and three field of views (320 μm × 320 μm) per section located ventral and ventromedial to the nucleus ambiguus were imaged. For embryos and neonates the “Atlas of the developing mouse brain: at E17.5, PO, and P6” (Paxinos et al., 2007) was used as reference. Counting of cells was performed using Zeiss ZEN software.

### Analysis of DNA Recombination in VIACO Mice

Genomic DNA from brain stem was isolated with the Invisorb Spin Tissue Mini Kit according to the manufacturer’s instructions (Stratec Biomedical, Birkenfeld, Germany). Two microliter of DNA was amplified using FastStart Taq DNA Polymerase (Roche, Mannheim) and the primers (250 nM final concentration each; 5′–3′ sequence) fwd: CCTTCTGGGTCCACACTTCTTCTA, rev: CCCCACTCGGCCCCTCATTGTA yielding a product of 522 bp after Cre mediated DNA recombination. As positive control PCR on the *Nex1* gene locus was performed (Primer fwd: AGAATGTGGAGTAGGGTGAC; rev: GAGTCCTGGAATCAGTCTTTTTC; product size 770 bp).

### Western Blot

Freshly isolated brain stem tissue was lysed in buffer A [50 mM Tris-HCl, pH 7.4; 150 mM NaCl; 1 mM EDTA; 1x Complete Protease Inhibitor cocktail (Roche, Mannheim, Germany)] using sonification. After centrifugation the pellet was resuspended in buffer B (50 mM Tris-HCl, pH 7.4; 150 mM NaCl; 1 mM EDTA; 1% Triton X-100; 0.1% SDS; 1x Complete Protease Inhibitor cocktail). 10 μg of protein mixed with 5x sample buffer were loaded on a 10% polyacrylamide gel and after electrophoresis blotted onto a nitrocellulose membrane. Membranes were blocked with 3% bovine serum albumin in 50 mM Tris/HCl, 150 mM NaCl, 0.2% Triton X-100 (pH 7.5) before they were incubated with the primary antibodies rabbit anti-VIAAT or mouse anti-beta-Actin (see Supplementary Table 1 for details on the antibodies) in 1% bovine serum albumin, 50 mM Tris/HCl, 150 mM NaCl, 0.2% Triton X-100 (pH 7.5). After six washing steps, blots were incubated with the secondary antibodies (Supplementary Table 1) and developed using the SuperSignal West Femto Chemiluminescent Substrate (Thermo Scientific) and imaged by an Intas Imaging System (Intas, Göttingen, Germany). Bands were quantified using Quantity One, Version 4.6.2 Basic (Bio-Rad, Hercules, CA, United States), and normalized the intensity of the beta-actin control of the same sample.

### Unrestrained Whole-Body Plethysmography

Mice could freely move in the plethysmography chamber (volume 1180 ml for adult mice, 50 ml for neonates) during the measurement. Whole-body plethysmography utilizes the pressure changes resulting from the warming of the inspired air and cooling during expiration (Drorbaugh and Fenn, 1955). For adult mice, we used the chamber in a flow-through configuration (Zhang et al., 2014; Hülsmann et al., 2016) with a positive bias airflow of 150 ml min^–1^. Breathing of neonates was measured without additional airflow. Mice were allowed to adapt to the plethysmography chamber for about 12 min prior to acquisition of the data used for analyzing the breathing rhythm. Pressure differences between the recording chamber and a reference chamber were captured by a DP103-12 pressure transducer (Validyne Engineering; sensitivity ± 0.02 psid full range) and passed through a sine wave carrier demodulator (CD-15, Validyne Engineering) for digitization (1 kHz sampling rate) with an analog-digital interface (Axon, MiniDigi 1B) and Axoscope software (Molecular Devices). Since chamber temperature and humidity was not measured, we did not perform corrections for these parameters and refrained from analysis of volume data. Prior to off-line analysis with LabChart software (AdInstruments), the raw signal was band pass filtered offline (3–30 Hz), to remove movement artifacts and noise. The peak detection module of LabChart was used to identify positive pressure peaks corresponding to inspiration. Respiratory rate (min^–1^) was calculated as the reciprocal of the averaged peak to peak interval of the inspiratory flow. All respiratory cycles were used, regardless of the underling behavior, e.g., sniffing or grooming. Intervals that where longer than 1 s were considered as pauses, calculated as pauses per minute. Irregularity scores (IrrScore) were calculated to assess the cycle-to-cycle variability of the interval (int) as IrrScoreInt = 100⋅|(Int(n)- Int(n-1))/Int(n-1)| (Barthe and Clarac, 1997; Wegener et al., 2014; Mesuret et al., 2018). To estimate the overall variability of the breathing, the coefficient of variation (CV) was calculated for respiratory cycle length (interval). Offline calculations were performed using Excel (Microsoft).

To discriminate between resting and behavioral (e.g., sniffing) breathing, the frequency of breathing was analyzed. Respiratory cycles were defined as behavioral breathing if the frequency was larger than 8 Hz (i.e., interval < 125 ms). To analyze breathing parameters of resting and behavioral breathing separately, the data was divided in time bins of 10 s and each time bin was classified as either resting or behavioral breathing using this threshold of 8 Hz (resting: < 10% breathing with more than 8 Hz; behavioral: > 50% breathing with more than 8 Hz; Ward et al., 2011). Time bins with 10% to 50% of breathing with more than 8 Hz were not analyzed further as they could not be assigned unequivocally. Within these time bins, respiratory parameters were analyzed as described above.

### Data Processing and Presentation

**FIGURE 1:**
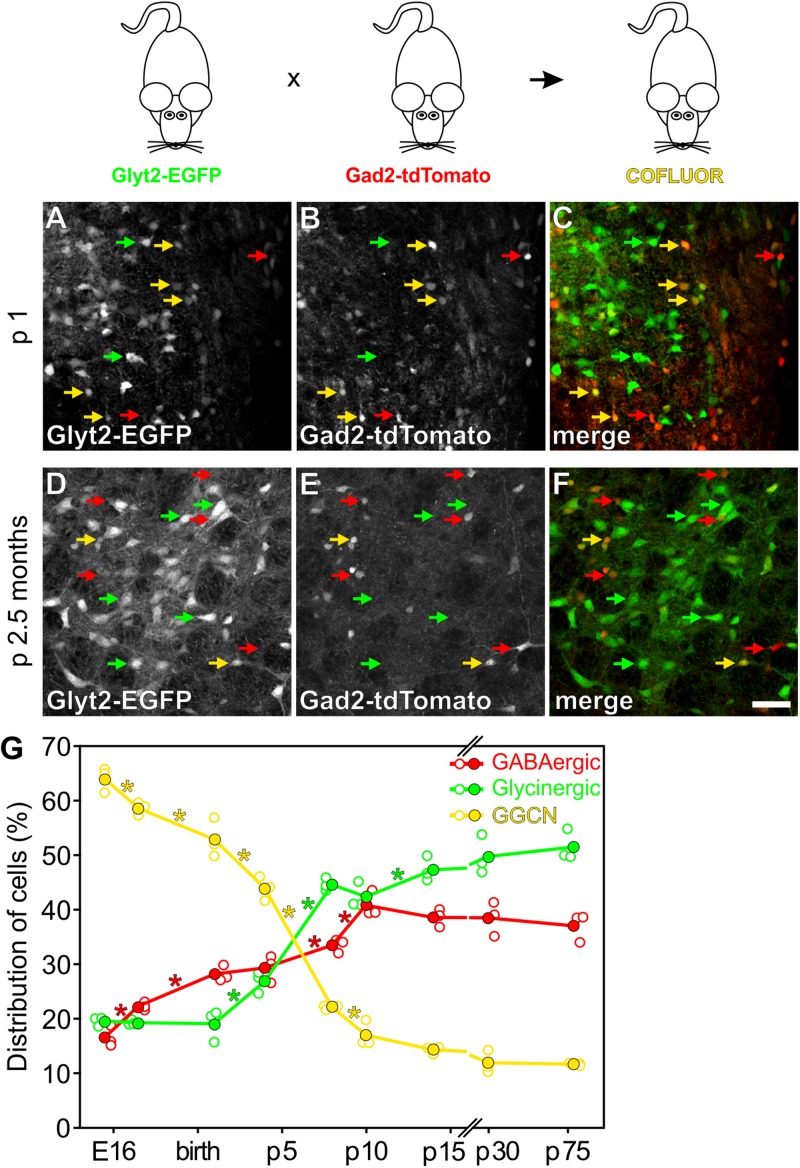
Identification of GGCN during development using COFLUOR mice. In COFLUOR mice, GGCN are highlighted by coexpression of EGFP (Glyt2-EGFP transgene) and tdTomato (Gad2-tdTomato transgene), while glycinergic and GABAergic neurons will be labeled by EGFP- or tdTomato expression, respectively. Examples of cell type identification at p1 **(A–C)** and 2.5 months **(D–F)**. **(C,F)** Overlay showing glycinergic neurons in green and GABAergic neurons in red. GGCN neurons appear in yellow due to coexpression of both fluorescent proteins. Scale bar in **(F)** corresponds to 50 μm and applies to **(A–F)**. **(G)** Quantification of the cell populations during development from embryonic day E15.5 to 2.5 months. Shown is the mean (filled circles) and the single data points (open circles) from *n* = 3 mice for each time point. In total, 7121, 5033, 4134, 2481, 2185, 2096, 1632, 1587, 1196 cells were counted for the time points from left to right, respectively. 100% corresponds to the sum of all three cell populations at each time point. ^∗^ Indicates significant difference between the two adjacent time points (*p* < 0.05, one way ANOVA).

**FIGURE 2:**
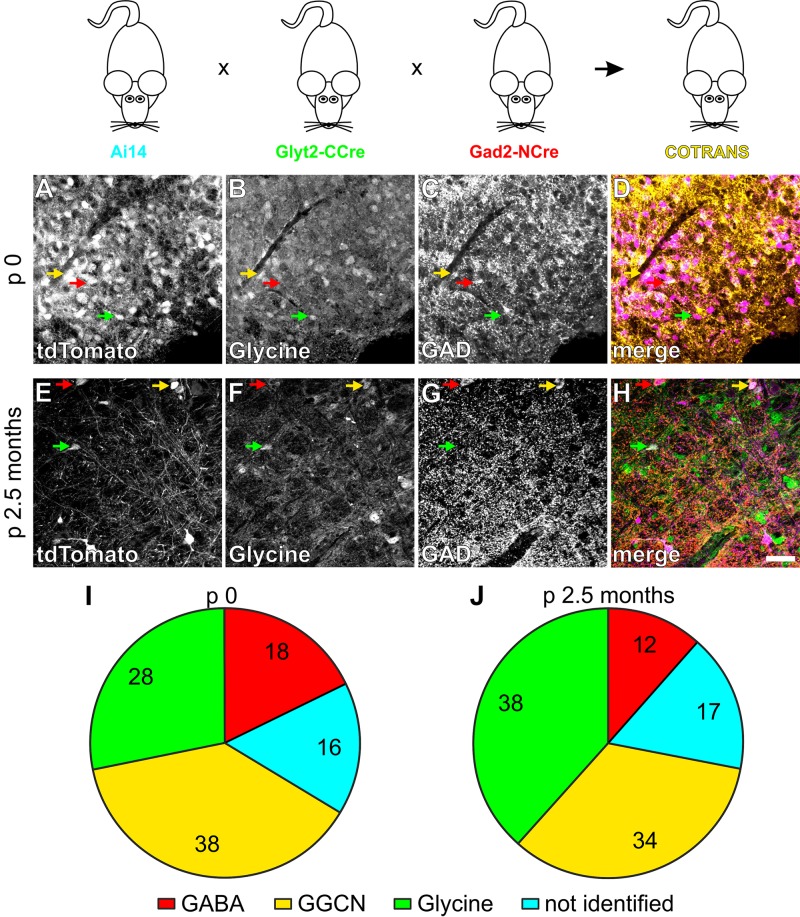
Fate mapping of individual GGCN using COTRANS mice. In COTRANS mice, GGCN, which have a GABA and glycine cotransmitting phenotype will be irreversibly labeled by tdTomato expression. Examples of fate identification at p0 **(A–D)** and 2.5 months **(E–H)**, by labeling tdTomato positive cells **(A,E)** for glycine **(B,F)** or GAD **(C,G)**. **(D,H)** Overlay with tdTomato in purple, glycine in green and GAD in red. Examples of GGCN neurons are marked with a yellow arrow. Scale bar in **(H)** corresponds to 50 μm and applies to **(A–H)**. Quantification of the cell populations at p0 **(I)** and 2.5 months **(J)**. Pie charts show the mean values of *n* = 3 and 8 mice for p0 and 2.5 months, respectively. In total, 1133 and 1109 cells were counted for p0 and 2.5 months of age, respectively. 100% corresponds to all cells labeled by tdTomato expression. See Supplementary Figure 4 for the distribution of data.

**FIGURE 3:**
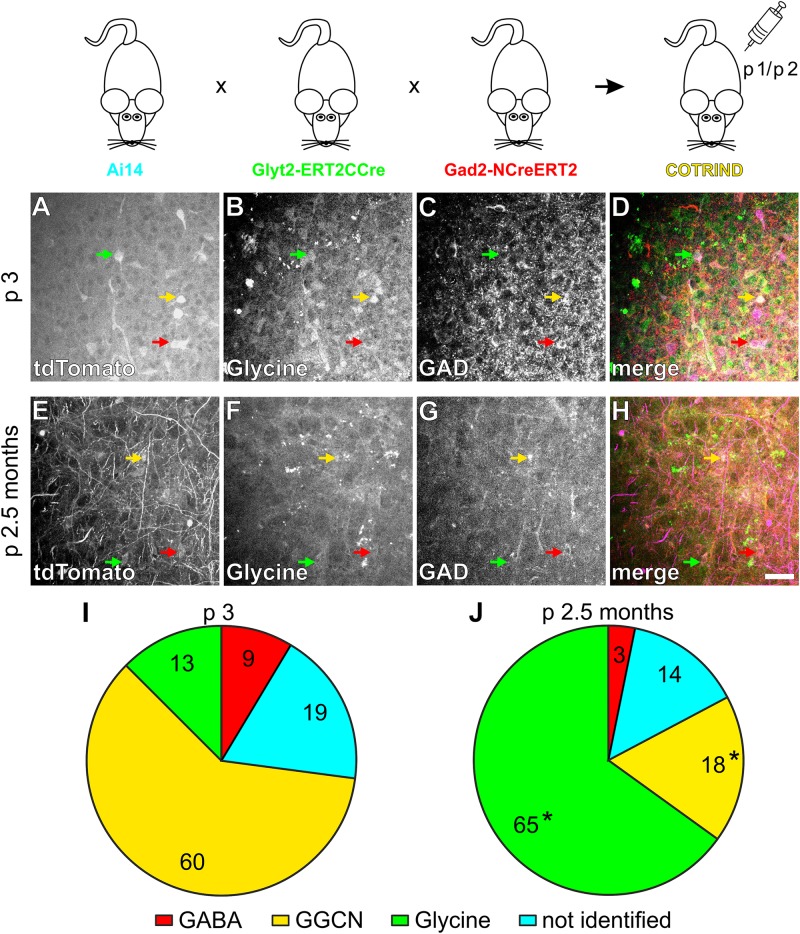
Fate mapping of individual GGCN present in the ventrolateral medulla at p1/p2 in COTRIND mice. In COTRIND mice, GGCN, which at the time of tamoxifen injection express a GABA and glycine cotransmitting phenotype will be irreversibly labeled by tdTomato expression. Mice were injected with tamoxifen at p1 and p2 and analyzed at p3 **(A–D,I)** or 2.5 months **(E–H,J)**. Examples of fate identification at p3 **(A–D)** and 2.5 months **(E–H)**, by labeling tdTomato positive cells **(A,E)** for glycine **(B,F)** or GAD **(C,G)**. **(D,H)** Overlay with tdTomato in purple, glycine in green and GAD in red. GGCN neurons are marked with a yellow arrow. Scale bar in **(H)** corresponds to 50 μm and applies to **(A–H)**. Quantification of the fate of the GGCN cell populations labeled at p1/p2 at p3 **(I)** and 2.5 months **(J)**. Pie charts in **(I,J)** show the mean values of *n* = 4 and 3 mice each for p3 and 2.5 months, respectively. In total, 238 and 148 cells were counted for p0 and 2.5 months of age, respectively. 100% corresponds to all cells labeled by tdTomato expression. See Supplementary Figure 5 for the distribution of data. ^∗^ In **(J)** indicates significant differences compared to the same cell type at p3 shown in **(I)** (*p* < 0.05; two way ANOVA).

**FIGURE 4:**
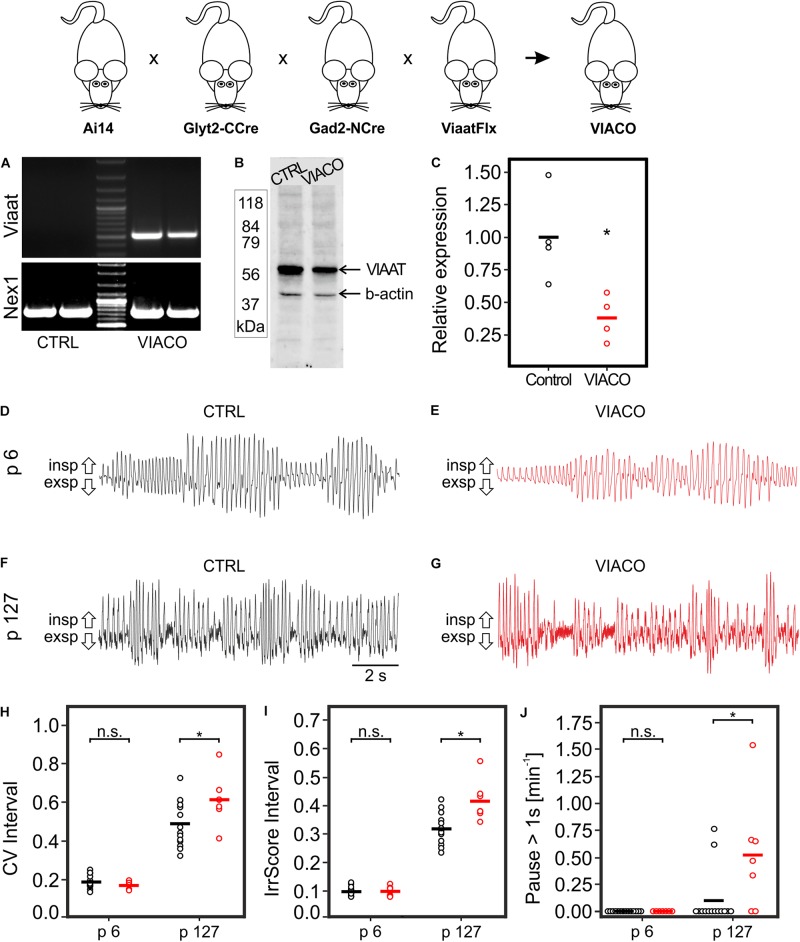
Breathing pattern in VIACO mice. COTRANS mice were crossed to mice with a LoxP-flanked Viaat allele (ViaatFlx), resulting in inactivation of GGCN neurons. **(A)** PCR showing recombination of the LoxP-sites within the Viaat gene in the brain stem of VIACO mice, but not in control mice. As positive control the unrelated *Nex1* gene locus was amplified. **(B)** Example of a Western blot showing reduction in VIAAT protein (55–60 kDa) in the brain stem in VIACO mice (p127) compared to control. β-actin (42 kDa) was used as a loading control. **(C)** The quantification of the Western blots shows a clear reduction in VIAAT protein in VIACO mice (p127; *n* = 4 for both genotypes; ^∗^*p* < 0.05, *t*-test). Breathing patterns of control mice **(D,F)** and VIACO mice **(E,G)** at the age of p6 **(D,E)** or p127 **(F,G)**. Quantification of the overall variability of the breathing **(H)**, the cycle-by-cycle variability **(I)** as well as the number of respiratory pauses (pause > 1s; **J**). *n* = 14, 7, 14, 7 mice for CTRL p6, VIACO p6, CTRL p127, and VIACO p127, respectively. Data of CTRL and VIACO mice is shown in black and red, respectively. ^∗^ Significant differences between genotypes, *p* < 0.05, two way ANOVA.

Microscopic images were processed using Zeiss Axiovision software, Zeiss ZEN software, Image J and Adobe Photoshop CS2. Pie charts and bar graphs were generated using Microsoft Excel and Sigma Plot. Statistical tests were performed using Sigma Plot [one way ANOVA with Student-Newman-Keuls *post hoc* test (Figure 1G); two way ANOVA, general linear model, Holm-Sidak multiple comparison (Figures 2I,J, 3I,J, 4H–J, Tables 2–4, Supplementary Figures 4, 5, and Supplementary Table 2); *t*-test, two sided (Figure 4C)]. No additional covariates were tested. All n numbers refer to the number of mice analyzed, which are considered as biological replicates. Scatter plots show the mean (line or filled circles) and all individual data points (open circles; Figures 1G, 4C,H–J and Supplementary Figures 2D,H,L,P 4, 5). Pie charts (Figures 2I,J, 3I,J) illustrate the mean; corresponding data of individual mice are shown in Supplementary Figures 4, 5. Final illustrations were arranged using Corel Draw X4 Graphic.

**TABLE 2:** Respiratory parameters of VIACO mice.

	**p6**		**p127**		
			
	**CTRL *n* = 14**	**VIACO *n* = 7**	**CTRL *n* = 14**	**VIACO *n* = 7**	**Test parameter**
Respiratory rate [min^–1^]	279.3 ± 35.1	291.3 ± 28.0	434.2 ± 80.8	424.4 ± 57.4	P_NT_ < 0.05; P_EVT_ 0.133; F_Age_ 58.669; P_Age_ < 0.001; F_GT_ 0.00339; P_GT_ 0.954; F_int_ 0.338; P_int_ 0.564
		§0.654	# < 0.001	§0.713, # < 0.001	
CV interval	0.19 ± 0.03	0.17 ± 0.02	0.49 ± 0.12	0.61 ± 0.13	P_NT_ < 0.05; P_EVT_ < 0.05; F_Age_ 162.62; P_Age_ < 0.001; F_GT_ 3.315; P_GT_ 0.001; F_int_ 5.94; P_int_ 0.020
		§0.665	# < 0.001	§0.005, # < 0.001	
IrrScore Interval	0.10 ± 0.02	0.10 ± 0.02	0.32 ± 0.05	0.42 ± 0.07	P_NT_ < 0.05; P_EVT_ < 0.05; F_Age_ 346.846; P_Age_ < 0.001; F_GT_ 11.377; P_GT_ 0.002; F_int_ 11.326; P_int_ 0.002
		§0.996	# < 0.001	§ < 0.001, # < 0.001	
Pause > 1s [min^–1^]	0 ± 0	0 ± 0	0.10 ± 0.25	0.52 ± 0.53	P_NT_ < 0.05; P_EVT_ < 0.05; F_Age_ 13.721; P_Age_ < 0.001; F_GT_ 6.378; P_GT_ 0.016; F_int_ 6.378; P_int_ 0.016
		§1.000	#0.314	§ < 0.001, # < 0.001	
CV Amplitude	0.41 ± 0.08	0.39 ± 0.07	0.26 ± 0.08	0.27 ± 0.07	P_NT_ < 0.05 P_EVT_ < 0.05; F_Age_ 31.713; P_Age_ < 0.001; F_GT_ 0.0808; P_GT_ 0.778; F_int_ 0.374; P_int_ 0.544
		§0.530	# < 0.001	§0.818, #0.004	
IrrScore Amplitude	0.24 ± 0.15	0.38 ± 0.53	0.25 ± 0.15	0.52 ± 0.75	P_NT_ < 0.05; P_EVT_ = 0.349; F_Age_ 0.337; P_Age_ 0.565; F_GT_ 2.670; P_GT_ 0.111; F_int_ 0.224; P_int_ 0.639
		§n.t.	#n.t.	§n.t., #n.t.	

*Data are shown as mean ± standard deviation (SD). Statistical analysis was performed with SigmaPlot 12.5 using two way ANOVA (General Linear Model; Degrees of Freedom: DF_*Total*_ 41) followed by Pairwise Multiple Comparison. P_*NT*_ = *P*-value normality test; P_*EVT*_ = *P*-value equal variance test; F_*Age*_ = *F*-value for comparison of age, F_*GT*_ = *F*-value for comparison of genotype; F_*int*_ = *F*-value for interaction; P_*Age*_ = *P*-value for comparison of age; P_*GT*_ = *P-*value for comparison of genotype; P_*Int*_ = *P*-value for interaction. Pairwise Multiple Comparison were performed using the Holm-Sidak method. *P*-values for comparison between genotype per age categories are marked as “§.” *P*-values for comparison between age categories are marked as “#.” n: number of animals tested from each genotype and age group. Apneas (Pause > 1 s) are calculated per minute. n.t.: not tested.*

**TABLE 3:** Sniffing cycles of VIACO mice.

	**p6**		**p127**		
			
	**CTRL *n* = 14**	**VIACO *n* = 7**	**CTRL *n* = 14**	**VIACO *n* = 7**	**Test parameter**
Sniffing cycles (%)	0.4 ± 0.5	0.7 ± 0.7	37.2 ± 17.1	41.9 ± 20.0	P_NT_ < 0.05; P_EVT_ < 0.05; F_GT_ 0.341; P_GT_ 0.563; F_Age_ 86.583; P_Age_ < 0.001; F_int_ 0.268; P_int_ 0.608
		§0.963	# < 0.001	§0.441, # < 0.001	

*Respiratory cycles with peak-to-peak interval of less than 0.125 ms (8 Hz) were analyzed. Data are shown as mean ± standard deviation (SD). Statistical analysis was performed with SigmaPlot 12.5 using two way ANOVA (General Linear Model; Degrees of Freedom: DFTotal 41) followed by Pairwise Multiple Comparison. P_*NT*_ = *P*-value normality test; P_*EVT*_ = *P*-value equal variance test; F_*Age*_ = *F*-value for comparison of age, F_*GT*_ = *F-*value for comparison of genotype; F_*int*_ = *F*-value for interaction; P_*Age*_ = *P*-value for comparison of age; P_*GT*_ = *P*-value for comparison of genotype, P_*Int*_ = *P*-value for interaction. Pairwise Multiple Comparison were performed using the Holm-Sidak method. *P*-values for comparison between genotype per age categories are marked as “§.” *P*-values for comparison between age categories are marked as “#.” n: number of animals tested from each genotype and age group.*

**TABLE 4:** Comparison of resting and behaviral breathing of VIACO mice (p127) (periods with maximal 10% vs. more than 50% behavioral breathing).

**Behavioral breathing**	**<10%**		**>50%**		
			
	**CTRL *n* = 13**	**VIACO *n* = 6**	**CTRL *n* = 12**	**VIACO *n* = 6**	**Test Parameter**
Respiratory rate [min^–1^]	321.1 ± 54.2	289.3 ± 40.0	524.7 ± 32.8	515.1 ± 11.8	P_NT_ 0.944; P_EVT_ < 0.05; F_GT_ 2.057; P_GT_ 0.161; F_Bhvl_ 220.895; P_Bhvl_ < 0.001; F_int_ 0.589; P_int_ 0.448
		§0.127	# < 0.001	§0.643, # < 0.001	
CV interval	0.30 ± 0.12	0.38 ± 0.06	0.52 ± 0.07	0.65 ± 0.08	P_NT_ < 0.05; P_EVT_ 0.665; F_GT_ 9.892; P_GT_ 0.004; F_Bhvl_ 61.024; P_Bhvl_ < 0.001; F_int_ 0.592; P_int_ 0.447
		§0.100	# < 0.001	§0.010, # < 0.001	
IrrScore Interval	0.27 ± 0.08	0.33 ± 0.05	0.36 ± 0.06	0.43 ± 0.06	P_NT_ < 0.05; P_EVT_ 0.918; F_GT_ 7.645; P_GT_ 0.009; F_Bhvl_ 15.981; P_Bhvl_ < 0.001; F_int_ 0.0563; P_int_ 0.814
		§0.081	# < 0.001	§0.043, # < 0.001	
Pause > 1s [min^–1^]	0.00 ± 0.00	0.01 ± 0.03	0.00 ± 0.25	0.03 ± 0.06	P_NT_ < 0.05; P_EVT_ 0.134; F_GT_ 6.661; P_GT_ 0.014; F_Bhvl_ 1.379; P_Bhvl_ 0.249; F_int_ 1.379; P_int_ 0.249
		§0.324	#1.00	§0.013, #0.162	
CV Amplitude	0.23 ± 0.06	0.27 ± 0.05	0.21 ± 0.06	0.26 ± 0.11	P_NT_ < 0.05; P_EVT_ 0.706; F_GT_ 3.091; P_GT_ 0.088; F_Bhvl_ 0.260; P_Bhvl_ 0.613; F_int_ 0.0621; P_int_ 0.805
		§n.t.	#n.t.	§n.t., #n.t.	
IrrScore Amplitude	0.22 ± 0.08	0.37 ± 0.39	0.25 ± 0.14	0.48 ± 0.61	P_NT_ < 0.05; P_EVT_ = 0.402; F_GT_ 3.403; P_GT_ 0.074; F_Bhvl_ 0.437; P_Bhvl_ 0.513; F_int_ 0.131; P_int_ 0.720
		§n.t.	#n.t.	§n.t., #n.t.	

*Time bins of respiration with less than 10% or more than 50% behavioral breathing (threshold 8 Hz) were analyzed separately. Data are shown as mean ± standard deviation (SD). Statistical analysis was performed with SigmaPlot 12.5 using two way ANOVA (General Linear Model; Degrees of Freedom: DFTotal 36) followed by Pairwise Multiple Comparison. P_*NT*_ = *P*-value normality test; P_*EVT*_ = *P*-value equal variance test; F_*Bhvl*_ = *F*-value for comparison of behavioral breathing, F_*GT*_ = *F*-value for comparison of genotype; F_*int*_ = *F*-value for interaction; P_*Bhvl*_ = *P*-value for comparison of behavioral breathing; P_*GT*_ = *P*-value for comparison of genotype; P_*Int*_ = *P*-value for interaction. Pairwise Multiple Comparison were performed using the Holm-Sidak method. *P*-values for comparison between genotype per behavioral categories are marked as “§.” *P*-values for comparison between behavioral categories are marked as “#.” n: number of animals tested from each genotype and behavioral (Bhvl) group. Apneas (Pause > 1 s) are calculated per minute. n.t.: not tested.*

## Results

To study the populations of GABAergic, glycinergic as well as GABA-glycine cotransmitting neurons (GGCN) within the respiratory network of the ventrolateral medulla, we took advantage of a double transgenic mouse line expressing the green fluorescent protein EGFP in glycinergic neurons and the red fluorescent protein tdTomato in GABAergic cells (Figure 1; referred to as “COFLUOR” mice; Zeilhofer et al., 2005; Besser et al., 2015). In these mice, GGCN can be readily identified by coexpression of both fluorescent proteins (Figures 1A–F). Quantification of the three populations of interneurons at different time points during development revealed that the proportion of GGCN continuously decreases from embryonic day E15.5 (64% of all labeled cells) until postnatal day p14 (14%; Figure 1G). The proportion of GABAergic cells started to increase at E17.5 (22%) and continued to rise until about p10. In contrast, the number of glycinergic cells was constant on a low level until after birth and only started to increase at p4, reaching a maximum at p8 (45%; Figure 1G). These data suggest that inhibitory neurons within the ventrolateral medulla are mostly generated as GGCN and then differentiate first into GABAergic and slightly later into glycinergic neurons. However, this population-based approach does not allow to unequivocally concluding whether a GGCN does indeed differentiate into a GABAergic or glycinergic phenotype or whether GGCN die and new GABAergic and glycinergic cells are born simultaneously. To address this issue, GGCN were irreversibly, genetically labeled by Cre-recombinase mediated DNA recombination taking advantage of the split-Cre-system and its tamoxifen inducible variant split-CreERT2 (Hirrlinger et al., 2009a, b). In brief, in this system two parts of Cre-DNA recombinase (NCre and CCre) are expressed under the control of two different promoters and DNA recombination only occurs if both parts are expressed in one cell at the same time (Hirrlinger et al., 2009a, b; Beckervordersandforth et al., 2010). Two mouse lines were generated, one expressing NCre in GABAergic neurons under the control of the Gad2-promoter, the second expressing CCre in glycinergic cells under the control of the Glyt2 promoter (Figure 2). In these mouse lines, 68% (mean, range 66 to 70%, *n* = 3) of all GABAergic cells in the preBötC express NCre and 68% (mean, range 61 to 80%, *n* = 3) of glycinergic neurons express the CCre protein (Supplementary Figures 2A–H). Importantly, only very few cells express the NCre or CCre, but do not show either a GABAergic or glycinergic phenotype, respectively (about 2% for both GABAergic and glycinergic neurons; Supplementary Figures 2D,H). This indicates that while not all GABAergic or glycinergic neurons are targeted by split-Cre expression, ectopic targeting of other cell types is rather negligible.

To study the fate of GGCN, these two mouse lines were crossbred to the Ai14 reporter mouse line expressing tdTomato after Cre-mediated DNA recombination (Madisen et al., 2010; Figure 2). In these triple transgenic mice (which are referred to as “COTRANS” mice in the following) neurons that have been GGCN at some point in their life, will be irreversibly labeled by tdTomato expression. Importantly, no expression of the tdTomato-reporter was observed in mice harboring only either the NCre- or CCre-allele (Supplementary Figure 3), indicating that cells indeed will only be labeled if both NCre and CCre are present. Analysis of the current phenotype of neurons was performed using immunohistochemistry staining GABAergic cells with antibodies for both GAD isoforms (GAD1 and GAD2) as well as glycinergic cells with an antibody detecting glycine. This analysis revealed that 18% of all cells labeled by tdTomato expression in mice at p0 are GABAergic, 28% are glycinergic and 38% are GGCN (Figures 2A–D,I and Supplementary Figure 4) suggesting that at birth, a large proportion of neurons, which were cotransmitting during embryonic development, had already changed their phenotype to either GABAergic or glycinergic. About 16% of tdTomato-expressing cells could not be allocated to either a GABAergic, glycinergic, or GGCN phenotype (Figure 2I and Supplementary Figure 4A).

At the age of 2.5 months 38% of all tdTomato expressing cells in COTRANS mice showed glycinergic properties (Figure 2J) albeit with a rather large variability (Range 15–58%; Supplementary Figure 4). In turn, the number of GGCN neurons decreased to 34% (Range 23–47%), and 12% of tdTomato expressing cells were identified as GABAergic cells, while 17% could not be allocated to one of the three phenotypes of interneurons (Figure 2J and Supplementary Figure 4B).

In split-Cre based COTRANS mice, recombination occurs in GGCN at any time during development and the labeling by tdTomato expression is irreversible. Therefore, the labeling of GGCN is integrated over time and this approach does not allow direct cell fate analysis of GGCN present at a certain time point during development. To address this issue, we took advantage of the inducible split-CreERT2 system (Hirrlinger et al., 2009a). In two additional mouse lines, the NCreERT2 protein was expressed under the control of the Gad2-promoter, while ERT2CCre was driven by the Glyt2 gene (Figure 3). Verification of the specificity of the transgene expression revealed that at p0 28% (mean, range 18 to 33%; *n* = 3) and 47% (mean, range 37 to 54%; *n* = 9) of all GABAergic and glycinergic cells, respectively, express the appropriate transgene within the ventrolateral medulla (Supplementary Figures 2I–P). Furthermore, similar to COTRANS mice, only a small minority of cells were found to express the transgene but could not be assigned to a certain neuronal phenotype (3 and 4% for GABA and glycine, respectively; Supplementary Figures 2L,P). Therefore, while only a rather small fraction of all GGCN neurons will be targeted due to the low penetrance of the transgene especially in the GABAergic cells, ectopic targeting of non-GGCN cells will be very unlikely.

These mouse lines were again crossbred to the Ai14 reporter mouse line, generating a triple transgenic mouse line, which will be referred to as “COTRIND” mice (for cotransmission inducible; Figure 3). These mice were injected with tamoxifen on p1 and p2 to induce labeling by tdTomato in neurons with a GGCN phenotype at this time point. Mice without application of tamoxifen did not show any labeled cells (data not shown). Analysis of the phenotype of the labeled cells one day later (p3) revealed that most (60%) of the labeled cells can be identified as GGCN as expected (Figure 3I and Supplementary Figure 5). The remaining cells show either a GABAergic or glycinergic phenotype or cannot be allocated to a specific phenotype (Figure 3I and Supplementary Figure 5). Based on the data obtained in COFLUOR mice (Figure 1) it is reasonable to assume, that several cells, which are GGCN just after birth and therefore labeled after tamoxifen injection, change their phenotype within the next day(s) to either an GABAergic or glycinergic phenotype.

Additionally, the phenotype of GGCN labelled on p1/p2 by tamoxifen injection in COTRIND mice was analyzed in mice at 2.5 months of age (Figures 3E–H,J). It was expected from the analysis of COFLUOR mice, that many of these cells will differentiated into a glycinergic phenotype; and, indeed, 65% of the tdTomato labeled cell population now is glycinergic. 18% of the cells remained GGCN, while the percentage of cells with a GABAergic phenotype slightly decreased in the period from p3 to 2.5 months (Figure 3J and Supplementary Figure 5). In summary, the combined fate mapping results obtained in COFLUOR- (Figure 1), COTRANS (Figure 2) as well as COTRIND- (Figure 3) mice provide strong evidence that (most) inhibitory neurons in ventrolateral medulla are born as GGCN and then differentiate first into GABAergic neurons and later into glycinergic cells.

To study the physiological, functional impact of GGCN and their descendants for the breathing rhythm in the preBötC, we aimed to specifically affect neurotransmission within GGCN. To this end, COTRANS mice were crossed to mice in which the gene for the vesicular inhibitory amino acid transporter (Viaat; official gene symbol Slc32a1) is flanked by LoxP sites, allowing cell-type specific deletion of this gene (referred to as ViaatFlx mice; Rahman et al., 2015). As the VIAAT protein is responsible for transporting both GABA and glycine into synaptic vesicles, deletion of this gene will prevent filling of synaptic vesicles in GGCN and thereby prevent signaling from these cells to other neurons without damaging the neurons themselves. This quadruple transgenic mouse line is referred to as “VIACO” mice in the following (for VIAAT conditional knock out; Figure 4). In those mice, recombination of the Viaat gene could readily be detected in the brain stem (Figure 4A), and reduction of VIAAT protein expression was observed using Western blotting (Figures 4B,C; full absence of VIAAT protein was not expected as the gene is only deleted in GGCN). Breathing of VIACO mice and littermate controls was measured in 6 day and 127 day old mice using whole-body plethysmography (Figures 4D–G). At p6, control mice and VIACO mice were indistinguishable regarding respiratory rate as well as the variability of the respiratory cycle (Table 2). However, VIACO mice at p127 developed a respiratory phenotype that was characterized by (1) an increase in the overall variability of the breathing (CV interval; VIACO 0.61 vs. CTRL 0.49; *p* < 0.001, two way ANOVA, Figure 4H); (2) an increase of the cycle-by-cycle variability (irregularity score of the interval, IrrScore Interval: VIACO 0.42 vs. CTRL 0.32; *p* < 0.001, two way ANOVA, Figure 4I) as well as (3) an increase of respiratory pauses (>1s; VIACO 0.52 min^–1^ vs. CTRL 0.10 min^–1^; *p* < 0.001, two way ANOVA, Figure 4J). However, no difference was detected for the respiratory rate (VIACO 424 min^–1^ vs. CTRL 434 min^–1^; Table 2).

The overall breathing rhythm is a composite of the resting breathing pattern and breathing related to behaviors like, e.g., sniffing with increased breathing frequency. To analyze whether the observed differences are related to breathing at rest or rather to behavioral breathing patterns, time intervals of 10 s were analyzed separately and classified as either resting or behavioral breathing (see methods for details). As almost no time intervals with behavioral breathing were detected in p6 mice (Table 3), the following analysis was restricted to p127 mice. Significant differences in parameters of respiration between VIACO and CTRL mice were only detected in time intervals with behavioral breathing patterns but not in time intervals with mainly resting breathing (Table 4), indicating that GGCN and their descendants might mainly contribute to fine control the rhythm of behavioral breathing patterns rather than controlling the generation of the basal respiratory rhythm.

Finally, also COTRIND mice were bred to the ViaatFlx mouse line yielding inducible Viaat conditional knockout animals (indVIACO). Mice were injected with tamoxifen at p1 and p2, and breathing was analyzed at the age of p6 and p127. However, no significant differences between the genotypes was detected (Supplementary Table 2), most likely due to the low penetrance of DNA recombination in GGCN neurons.

## Discussion

Breathing is an essential process requiring lifelong generation of its rhythm and adaptation of this rhythm to the organism’s needs. Generation of this crucial rhythmic neuronal activity and its modulation by internal and external cues requires interaction of numerous neurons, both excitatory and inhibitory, in the respiratory centers of the brain stem including the preBötC. We studied the cellular fate of a specific type of inhibitory neurons with a GABA and glycine cotransmitting phenotype (GGCN) as well as the functional relevance of GGCN and their descendants. We find that most inhibitory neurons in the ventrolateral medulla develop through a GGCN stage. Furthermore, functional interference with these neurons results in subtle disturbance of the breathing rhythm, suggesting that these neurons are responsible for fine-tuning breathing. Finally, to our knowledge this is the first demonstration of the functionality of the split-CreERT2 system in transgenic mice *in vivo*. In addition, while different types of neurons have been targeted using other combinatorial genetic systems before (Kim et al., 2009; Taniguchi et al., 2011; Madisen et al., 2015; Okaty et al., 2015; He et al., 2016; Hennessy et al., 2017), the current study is the first report of targeting interneurons by the split-Cre system.

### Technical Considerations

The transgenic mouse lines used are powerful tools to study the cellular phenotype as well as its history; however, these technologies have some inherent limitations. First, in all mouse lines used, not all cells of the targeted cell type express the transgene (Zeilhofer et al., 2005; Besser et al., 2015). While this is often an issue in transgenic mouse lines, this limitation is even exacerbated in COFLUOR, COTRANS and COTRIND mice as the probability to hit a cotransmitting neuron is the product of the probability of each transgene (due to independent patterns of expression of both the Gad2- and Glyt2-transgenes). Consequently, only a subpopulation of each cell type was accessible for analysis and it cannot be fully excluded that this subpopulation is not representative of the whole population. While in COFLUOR mice a rather high proportion of GABAergic or glycinergic cells are targeted by expression of the fluorescence protein (84 and >90%, respectively; Zeilhofer et al., 2005; Besser et al., 2015), the penetrance of the transgenes is lower in case of COTRANS and COTRIND mice (Supplementary Figure 2). Therefore, the rather high proportion of GGCN in COTRANS mice at 2.5 month of age combined with the very different probability of targeting a GGCN (COFLUOR: 84% × 90% = 75%; COTRANS: 68% × 68% = 46%; COTRIND: 28% × 47% = 13%) might imply that the expression of split-Cre-transgenes in this mouse line shows a bias toward GGCN neurons. Importantly though, only very few cells expressed the transgene in the absence of the appropriate phenotype in all mouse lines (Zeilhofer et al., 2005; Besser et al., 2015; Supplementary Figure 2). Therefore, expression of the transgenic marker proteins is specific for the cell type, but does not include all cells of a given phenotype.

In addition, a direct comparison of the data obtained with the three different transgenic approaches is complicated by the fact that the cell population, which constitutes 100% of the analyzed cells, is different in these lines. While in COFLUOR mice, the sum of GGCN + glycinergic + GABAergic cells at the time point of analysis reflects the total number of cells, in COTRANS mice the number of tdTomato labelled cells (i.e., cells which have been GGCN at any point during development) is integrated over time. Finally, in the COTRIND mouse line tdTomato expressing cells reflect GGCN neurons at the time of tamoxifen injection.

When interpreting the data, one has to consider the intrinsic impreciseness of the transgenic tools in time. After an individual cell starts to express a certain phenotype reflected by activation of the respective promoter, it takes some time to express the fluorescent or the split-Cre protein. Vice versa, after a promoter is switched off, the loss of the protein depends on protein degradation, which also takes time and might be different for the different marker proteins. This results in some “time smearing” of the analysis of cell fate development implying that statements on the time point of cell fate changes require some caution. This time delay in degradation of split-CreERT2 proteins might provide an additional explanation for the observation that in COTRIND mice injected with tamoxifen at p1/p2 only about 60% of labeled cells are GGCN neurons at p3. In these cells, the transition to a GABAergic or glycinergic phenotype might have started already prior to birth, but the split-CreERT2 protein might not have been fully degraded by p1. Nevertheless, the overall change in the cell population as well as the change in the phenotype of individual labeled cells will be well reflected by this transgenic approach.

Finally, a number of cells labeled genetically by split-Cre induced tdTomato expression could not be allocated to either a GABAergic, glycinergic, or GGCN phenotype. The reason for this observation might be twofold: first, GGCN neurons might transit to other (i.e., non-GABAergic – non-glycinergic) neuronal phenotypes. Second, limitations of the antibodies and immunohistochemical methods used to identify the current phenotype of neurons might result in failure to identify the phenotype of these cells. Nevertheless, when taking this technical consideration and caveats into account, the analysis of the transgenic mouse lines described in this work provides a powerful tool to study the development, phenotype, fate and function of inhibitory neurons.

### Development and Physiology

In this paper, three different approaches were used to investigate the development of the phenotype of inhibitory neurons in the ventrolateral medulla: in COFLUOR mice, the current phenotype of a cell is visualized by expression of fluorescent proteins. In COTRANS mice, cells are labeled irreversibly if they once in their lifetime expressed a cotransmitting phenotype. Finally, using the COTRIND mouse line, GGCN can be labeled at a specific point in time allowing to track the fate of GGCN that were present at this time point. These combined experiments provide strong evidence that most inhibitory neurons in the ventrolateral medulla first show a cotransmitting phenotype. In COFLUOR mice, 53% of all labeled inhibitory neurons were identified as GGCN at birth, very similar to the rat preBötC at p1-3 (Koizumi et al., 2013) and confirming previous data showing that many glycinergic neurons also express markers for a GABAergic phenotype in early postnatal mice (Rahman et al., 2013). Around birth, cells start to differentiate in a first wave to GABAergic neurons, and differentiation to a purely glycinergic phenotype starts a few days later. While the respiratory network has to be functional immediately after birth, in the auditory system a shift from GABAergic to glycinergic inhibitory transmission was described around hearing onset (Nerlich et al., 2017), suggesting that these changes in properties of inhibitory neurons are associated with the maturation of the respective neuronal networks.

Deletion of Viaat in glycinergic neurons is lethal shortly after birth (Rahman et al., 2015) while mice with deletion of Glyt2 in glycinergic neurons survive until the second postnatal week (Gomeza et al., 2003). While this surprising observation might be explained by the pronounced, but incomplete loss of glycinergic transmission in Glyt2 knockout mice (Gomeza et al., 2003), the observed differentiation of inhibitory neurons within the respiratory network provides another conclusive explanation: deletion of Viaat removes both GABAergic and glycinergic transmission, whereas deletion of Glyt2 only affects glycinergic transmission. Since GABAergic transmission is normal in Glyt2 knockout mice (Latal et al., 2010), GABA mediated signals might provide sufficient inhibition as long as many neurons are still cotransmitting. Later, when the major transition of cotransmitting neurons to glycinergic neurons takes place toward the end of the first postnatal week, diminished inhibition by glycinergic transmission will become functionally more relevant. However, as besides the respiratory network in the preBötC also motor functions are impaired, it is unlikely that Glyt2-knockout mice die due to a direct failure of the central respiratory network (Hülsmann et al., 2019).

When neurotransmission of GGCNs and their descendants was impaired by conditional deletion of Viaat, the mice rather unexpectedly showed an overall normal breathing pattern at birth. The reason for this finding might be twofold: first, as discussed above most likely a maximum of 50% of cotransmitting neurons will be targeted by the split-Cre approach and it is likely that the other 50% might be sufficient for maintaining a rather normal breathing phenotype. Consistent with these assumptions, no breathing abnormalities were detected in inducible VIACO mice injected with tamoxifen on p1/p2. As the transgene penetrance is even lower in these mice (see above) and they do not integrate GGCN over time, most likely even fewer cells are hit by the genetic manipulation in this mouse line. Secondly, there is a long standing and so far unresolved debate whether inhibitory neurons are indeed crucial for generation of the breathing rhythm, or whether mainly excitatory neurons generate the rhythm which is modulated by inhibitory neurons (Sherman et al., 2015; Marchenko et al., 2016; Cregg et al., 2017; Ausborn et al., 2018; Baertsch et al., 2018). While we cannot formally exclude that the sole reason for the observed phenotype is the first point, our results are in line with the concept that the breathing rhythm is mainly generated by excitatory neurons and modulated by inhibitory neurons. Consistently, VIACO mice show an increased variability of breathing and increase number of apnea episodes, which is typically seen in pathological situation with partial impairment of the function of inhibitory neurons (Chao et al., 2010; Hülsmann et al., 2016).

In summary, using a set of novel mouse genetic tools we characterized the cellular relation of inhibitory neurons in the respiratory network of the ventrolateral medulla with different neurotransmitter phenotypes and addressed their functional relevance for the modulation of the rhythmic neuronal activity underlying breathing. We observe a developmental shift from GGCN to GABAergic and glycinergic neurons, as well as an increased variability of the breathing rhythm if transmission is impaired in GGCN. These findings provide new insights into the properties of the neuronal network in the respiratory center and how this network is fine-tuned and modulated for achieving lifelong reliable function.

## Data Availability Statement

All datasets generated for this study are included in the article/Supplementary Material.

## Ethics Statement

The animal study was reviewed and approved by the Animal Welfare Office of the Faculty of Medicine, University of Leipzig and Governmental Authorities of Saxony (Landesdirektion Sachsen) or Animal Welfare Office and Commission of the University Medical Center Göttingen and Governmental Authorities of Lower Saxony (LAVES).

## Author Contributions

JH and SH conceived and supervised the project. GM, SB, MS, PH, and UW acquired the data. JH, GM, SB, SK, PH, VE, UW, and SH analyzed and interpreted the data. SW provided essential tools. JH, SK, and SH wrote the manuscript with input of all co-authors. All authors approved the final version of the manuscript.

## Conflict of Interest

The authors declare that the research was conducted in the absence of any commercial or financial relationships that could be construed as a potential conflict of interest.
