# Supplementary material for: GABA-Glycine Cotransmitting Neurons in the Ventrolateral Medulla: Development and Functional Relevance for Breathing
Source: Front Cell Neurosci. 2019 Nov 19;13:517. doi: 10.3389/fncel.2019.00517 (PMC6877658; doi:10.3389/fncel.2019.00517)
Supplement: Supplementary file 1 [file Data_Sheet_1.pdf]

Supplementary Material

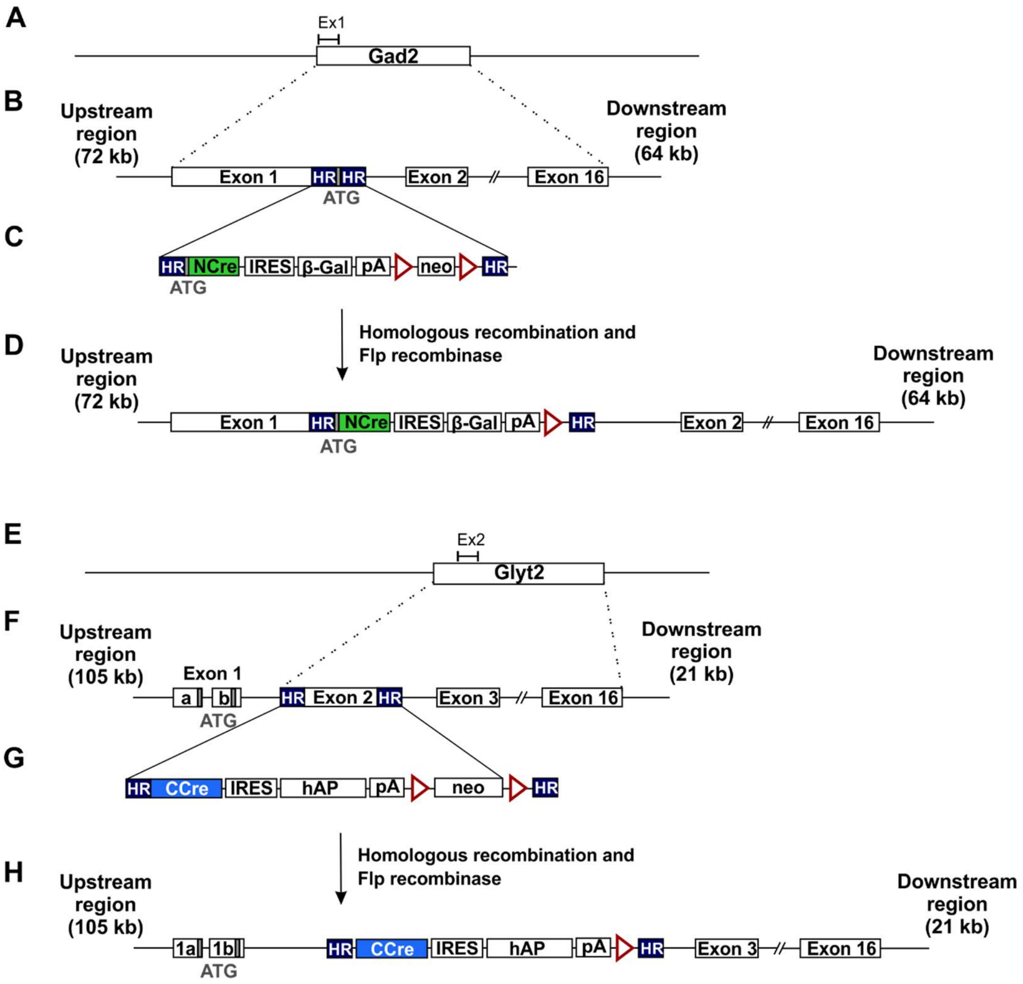

Suppl. Fig. 1: Generation of Split-Cre and Split-CreERT2 transgenic mice targeting inhibitory neurons. (A-D) Generation of the Gad2 transgene constructs. (**A)** BAC clone RPCI23-407K8 contains the entire mouse Gad2 gene. (**B)** Homology regions (HR) bordering the ATG start codon of the Gad2 gene were selected to inserted the targeting construct **(C)** containing the NCre open reading frame (ORF) followed by an IRES, the β-Gal coding sequence and a SV40 poly-adenylation site (pA). After homologous recombination, the Frt site (triangles) flanked neomycin selection cassette was removed by Flp mediated recombination yielding the final transgene construct **(D)**. To generate the Gad2-NCreERT2 transgene construct, the same strategy was used, but the NCre ORF was replaced by the NCreERT2 ORF. (**E-H)** Generation of the Glyt2 transgene constructs (official gene symbol Slc6a5). **(E)** BAC clone RP23-365E4 contains the whole mouse Glyt2 gene. **(F)** Homology regions (HR) were selected within exon 2 of the Glyt2 gene to insert the targeting construct **(G)** containing the CCre ORF followed by an IRES, the coding sequence for human alkaline phosphatase (hAP) and the pA. The neomycin selection cassette was removed as above generating the final transgene construct **(H)**. To generate the Glyt2-ERT2CCre transgene construct, the same strategy was used, but the CCre ORF was replaced by the ERT2CCre ORF.

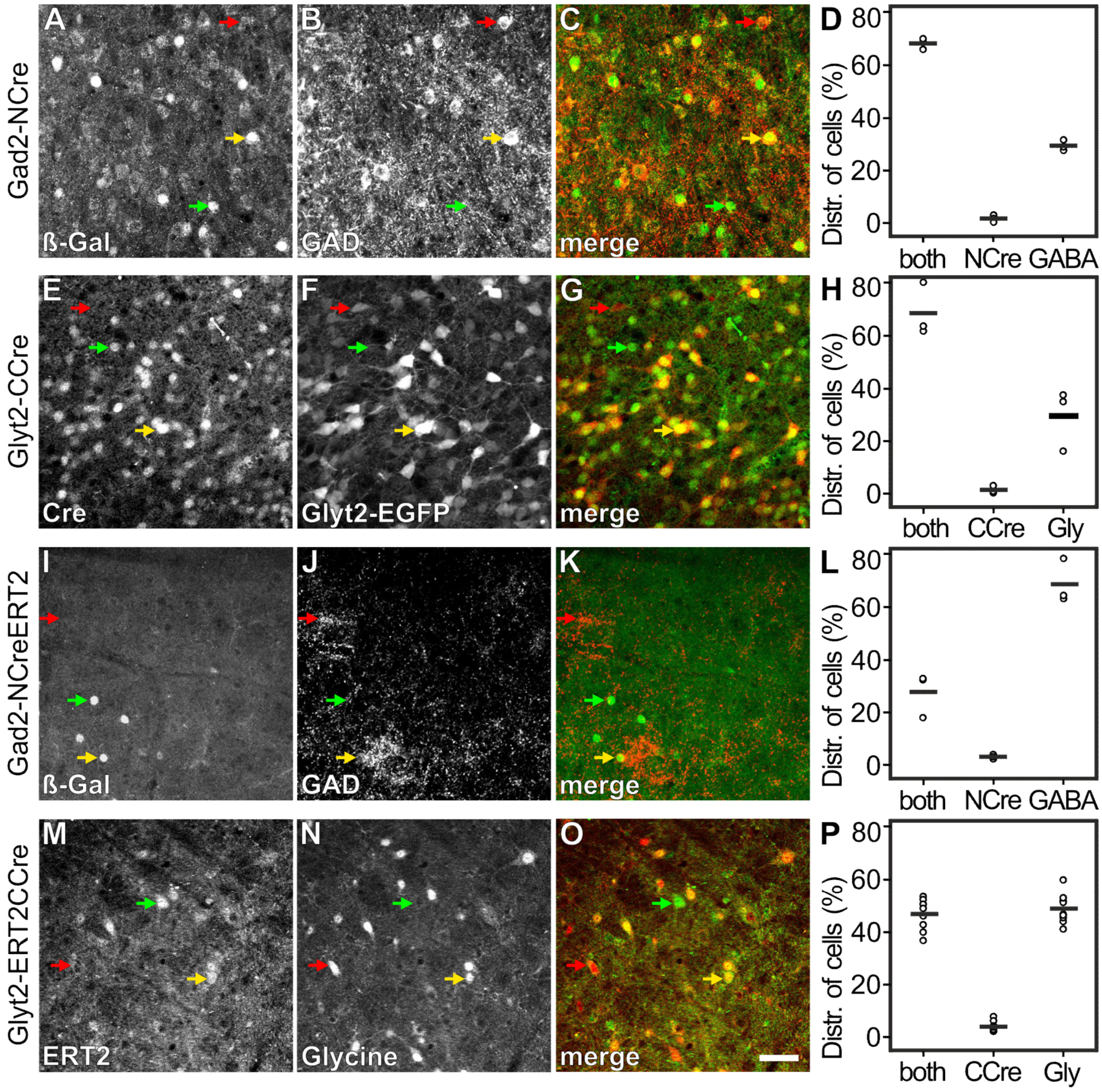

Suppl. Fig. 2: Verification of the specificity of the expression of split-Cre proteins. To characterize the four novel split-Cre(ERT2) transgenic mouse lines targeting GABAergic and glycinergic neurons, a transgene specific marker (NCre, NCreERT2: β-Gal (A, I); CCre: Cre (E); ERT2CCre: ERT2 (M)) was labelled with an appropriate antibody while the GABAergic or glycinergic phenotype was identified using immunhistochemical staining for GAD2 (B, J), EGFP expression in Glyt2-EGFP mice (F) or immunhistochemical staining for glycine (N), respectively. The overlays (C, G, K, O) show the transgene marker in green, the cell type marker in red and cells positive for both markers in yellow. Panels D, H, L, P show the quantification of cells expressing both the transgene marker and the corresponding cell type marker (both), only the transgene marker (NCre, CCre) or only the cell type marker (GABA, Gly). Importantly, only very few cells express only the transgene marker, which implies that transgene expression is specific for the targeted cell type. Analysis was performed on n = 3, 3, 3, 9 mice for the Gad2-NCre, Glyt2-CCre, Gad2-NCreERT2 and Glyt2-ERT2CCre mouse lines, respectively. 527, 2783, 505 and 948 cells were counted in total for the Gad2-NCre, Glyt2-CCre, Gad2-NCreERT2 and Glyt2-ERT2CCre mouse lines, respectively. The scale bar in O corresponds to 50 µm and applies to all panels.

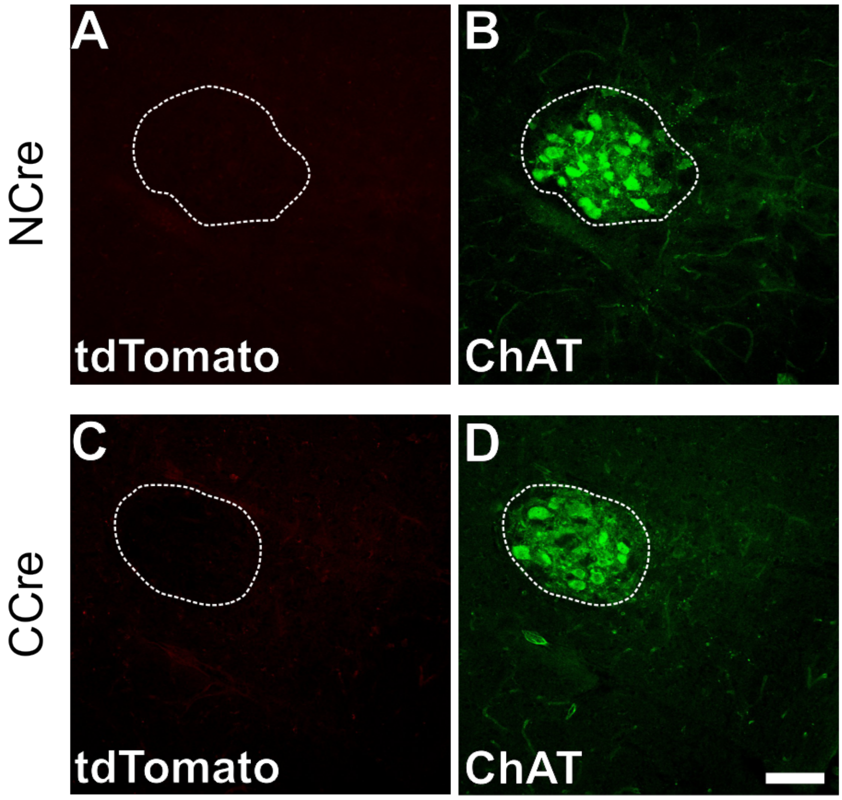

Suppl. Fig. 3: No reporter expression is observed in mice harboring only one of the split-Cre alleles. To test for the specificity / leakiness of split-Cre mediated DNA recombination, mice harboring (in addition to the Ai14 reporter allele) only either the Gad2-NCre (A, B) or the Glyt2-CCre allele (C, D) were analyzed for expression of the tdTomato reporter protein (A, C). However, no reporter expression was observed. Slices were costained with an antibody against choline acetyltransferase (ChAT; B, D), which labels the nucleus ambiguus, which is next to the analyzed region of the ventrolateral medulla including the preBötzinger complex. Shown are examples of a 174 days and 149 days old mouse for NCre and CCre, respectively. As these mice are older than the other mice analyzed in this study and DNA recombination is irreversible (i.e. tdTomato$^+$ cells will accumulate over time) this result strongly suggests that no DNA recombination occurs in mice containing only either the Gad2-NCre or the Glyt2-CCre allele. Scale bar: 100 µm.

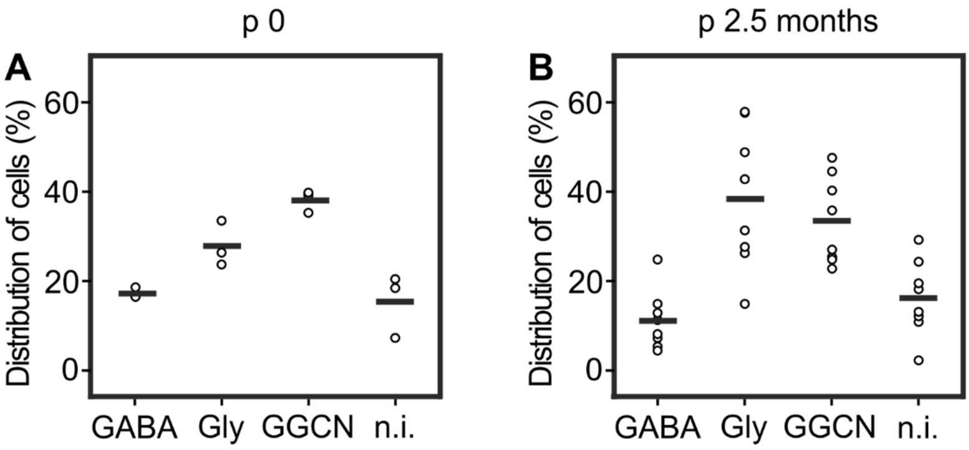

Suppl. Fig. 4: Fate mapping of individual GGCN using COTRANS mice. Shown are data of all individual mice analyzed at p0 **(A)** and 2.5 months of age **(B)** (n = 3 and 8 mice for p0 and 2.5 months, respectively) as well as the mean value (line) which is also shown in the pie charts in Fig. 2. Total number of cells (100 %) corresponds to all cells labelled by tdTomato expression. Percentage of tdTomato$^+$ cells showing a pure GABAergic (GABA), a pure glycinergic (Gly) or a GABA and glycine cotransmitting phenotype (GGCN) is shown. n.i.: percentage of tdTomato$^+$ cells which could not be allocated to one of these three phenotypes.

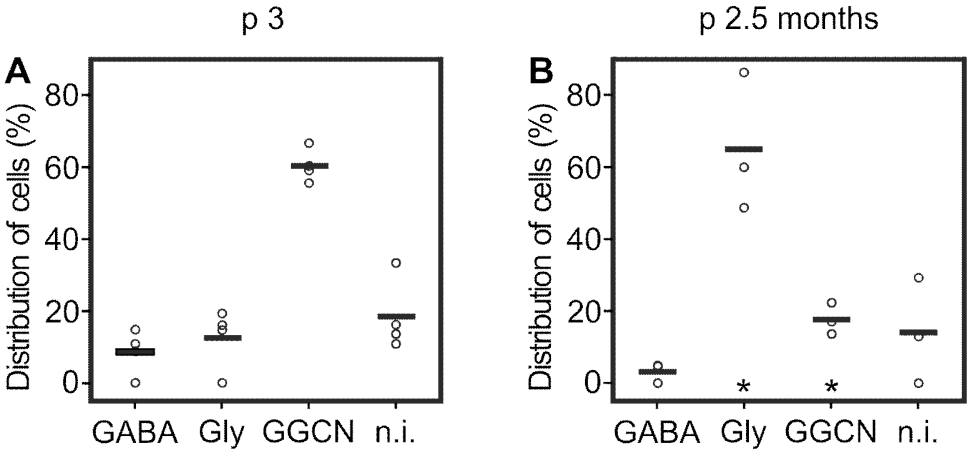

Suppl. Fig. 5: Fate mapping of individual GGCN using COTRIND mice. COTRIND mice were injected with tamoxifen at p1/p2 and analyzed at the age of p3 **(A)** and 2.5 months **(B)**. Shown are data of all individual mice analyzed (n = 4 and 3 for p3 and 2.5 months, respectively) as well as the mean value (line) which is also shown in the pie charts in Fig. 3. Total number of cells (100 %) corresponds to all cells labelled by tdTomato expression. Percentage of tdTomato$^+$ cells showing a pure GABAergic (GABA), a pure glycinergc (Gly) or a GABA and glycine cotransmitting phenotype (GGCN) is shown. n.i.: percentage of tdTomato$^+$ cells which could not be allocated to one of these three phenotypes. * in B indicates significant differences compared to the same cell type at p3 shown in A ($p < 0.05$; two way ANOVA).

Supplementary Table 1: Antibodies used in this study

Target	Antibody	dilution	Supplier	Catalogue number	Lot
glycine	rat anti-glycine	1:1000 (IHC)	Immunosolutions	IG1002	1131
Gad1	mouse anti-GAD1	1:1000 (IHC)	Millipore	MAB5406	2491208
Gad2	mouse anti-GAD2	1:500 (IHC)	Millipore	MAB351	LV1579927
Cre	mouse anti-Cre Recombinase	1:1000 (IHC)	Millipore	MAB3120	2387475
β-Gal	rabbit anti-ß-Gal	1:2000 (IHC)	MP Biomedicals	855976	
ERT2	rabbit anti-ERα HC-20	1:1000 (IHC)	SantaCruz	sc-543	
ChAT	goat anti-Cholinacetyl-transferase	1:500 (IHC)	Millipore	AB144	3067431
VIAAT	rabbit anti-VIAAT	1:3000 (WB)	SynapticSystems	131 003	131003/38
β-actin	mouse anti-beta-Actin	1:1000 (WB)	Sigma-Aldrich	A5441	030M4788
	goat anti-rabbit-IgG Cy3	1:1000 (IHC)	DIANOVA	111-165-144	75692
	goat anti-mouse-IgG Cy5	1:1000 (IHC)	DIANOVA	115-175-166	72032 & 121792
	goat anti-rat-IgG AlexaFluor488	1:1000 (IHC)	Invitrogen	A-11006	93C1-1
	donkey anti-goat-IgG AlexaFluor488	1:500 (IHC)	DIANOVA	705-545-147	103943
	goat anti-rabbit-IgG HRP	8ng/ml (WB)	Jackson ImmunoResearch	111-035-003	63590
	goat anti-mouse-IgG HRP	8ng/ml (WB)	Jackson ImmunoResearch	115-035-003	72639

IHC: Immunohistochemistry; WB: Western Blot

Supplementary Table 2: Respiratory parameters of mice with tamoxifen inducible deletion of VIAAT in GGCN.

	p6		p127		
	CTRL n=10	indVIACO n=9	CTRL n=16	indVIACO n=11	Test parameter
Respiratory rate [min^{-1}]	309.4 ± 13.7	307.4 ± 20.3 §0.932	411.4 ± 69.8 #<0.001	394.3 ± 53.1 §0.390, #<0.001	P_{NT} < 0.05; P_{EVT} < 0.05; F_{Age} 38.703; P_{Age} <0.001; F_{GT} 0.395; P_{GT} 0.533; F_{int} 0.248; P_{int} 0.621
CV interval	0.17 ± 0.04	0.15 ± 0.03 §0.503	0.43 ± 0.09 #<0.001	0.44 ± 0.13 §0.644, #<0.001	P_{NT} 0.578; P_{EVT} < 0.05; F_{Age} 110.986; P_{Age} <0.001; F_{GT} 0.0453; P_{GT} 0.833; F_{int} 0.666; P_{int} 0.419
IrrScore Interval	0.10 ± 0.03	0.08 ± 0.02 §0.692	0.29 ± 0.06 #<0.001	0.32 ± 0.10 §0.306, #<0.001	P_{NT} 0.150; P_{EVT} < 0.05; F_{Age} 125.875; P_{Age} <0.001; F_{GT} 0.136; P_{GT} 0.714; F_{int} 0.952; P_{int} 0.335
Pause > 1s [min^{-1}]	0 ± 0	0 ± 0 §n.t.	0.04 ± 0.11 #n.t.	0.09 ± 0.21 §n.t., #n.t.	P_{NT} < 0.05; P_{EVT} < 0.05; F_{Age} 3.100; P_{Age} 0.086; F_{GT} 0.409; P_{GT} 0.526; F_{int} 0.409; P_{int} 0.526
CV Amplitude	0.34 ± 0.04	0.29 ± 0.09 §n.t.	0.27 ± 0.21 #n.t.	0.24 ± 0.04 §n.t., #n.t.	P_{NT} < 0.05; P_{EVT} < 0.05; F_{Age} 2.072; P_{Age} 0.157; F_{GT} 1.172; P_{GT} 0.285; F_{int} 0.0269; P_{int} 0.870
IrrScore Amplitude	0.19 ± 0.11	0.13 ± 0.05 §n.t.	0.20 ± 0.10 #n.t.	0.20 ± 0.05 §n.t., #n.t.	P_{NT} < 0.05; P_{EVT} 0.661; F_{Age} 1.959; P_{Age} 0.169; F_{GT} 1.419; P_{GT} 0.240; F_{int} 1.216; P_{int} 0.276

COTRIND mice were bred to ViaatFlx mice resulting in inducible Viaat conditional knockout animals (indVIACO). After induction of DNA recombination by tamoxifen application at p1/p2, breathing patterns were analyzed at the age of p6 and p127 using whole-body plethysmography. Data are shown as mean ± standard deviation (SD). Statistical analysis was performed with SigmaPlot 12.5 using two way ANOVA (General Linear Model; Degrees of Freedom: DF_{Total} 45) followed by Pairwise Multiple Comparison. P_{NT} = P-value normality test; P_{EVT} = P-value equal variance test; F_{Age} = F-value for comparison of age, F_{GT} = F-value for comparison of genotype; F_{int} = F-value for interaction; P_{Age} = P-value for comparison of age; P_{GT} = P-value for comparison of genotype; P_{Int} = P-value for interaction. Pairwise Multiple Comparison were performed using the Holm-Sidak method. P-values for comparison between genotype per age categories are marked as "§". P-values for comparison between age categories are marked as "#". n: number of animals tested from each genotype and age group. Apneas (Pause > 1 s) are calculated per minute. n.t.: not tested.
